# Traumatic Acetabular Protrusion

**DOI:** 10.5811/cpcem.2018.3.37750

**Published:** 2018-05-18

**Authors:** William Weber, Jacob Moore, Navneet Cheema

**Affiliations:** The University of Chicago, Department of Medicine, Section of Emergency Medicine, Chicago, Illinois

## CASE PRESENTATION

A 69-year-old woman with a history of osteopenia and left total hip arthroplasty three months prior presented from home to the emergency department with leg pain and inability to ambulate. She had fallen from standing onto a tile floor, making contact with her left hip. She was mildly hypertensive, with a blood pressure of 137/92 mmHg and tachycardic, with a heart rate of 105 beats per minute, but had otherwise unremarkable vitals. On examination, she had tenderness and developing ecchymosis over the greater trochanter of the left femur. Her left leg was slightly shortened and externally rotated but neurovascularly intact. A pelvic radiograph (Image) showed medial displacement of the acetabulum and femoral head into the lesser pelvis. Angiography failed to reveal any vascular disruption. She remained hemodynamically stable and was taken to surgery for an urgent but successful internal pelvic fixation.

## DIAGNOSIS

Acetabular protrusion most commonly occurs as a chronic erosion of the acetabulum in patients with osteoporosis or other disease of the bone (e.g., osteomalacia, ankylosing spondylitis). Also, as in the case of acute trauma, force transmitted through joint can cause failure of acetabular integrity. This acetabular protrusion brings the femoral head in close proximity to pelvic contents, risking further injury. Providers should assess for sciatic nerve damage, which occurs in up to 30% of acute acetabular fractures, as well as injury to the iliac artery, which is at risk of shearing injury as it courses along the internal pelvic cavity.^2^ Unlike with other pelvic fractures, there is no role for pelvic binding in acetabular protrusion, as it can potentially worsen neurovascular damage.^3^ For the hemodynamically unstable patient, interventional radiology should be consulted for embolization of the internal iliac.^4^ Stable patients may be placed in traction to minimize medial force on the acetabulum.

Documented patient informed consent and/or Institutional Review Board approval has been obtained and filed for publication of this case report.

**CPC-EM Capsule**

What do we already know about this clinical entity?

Acetabular protrusion is a medial displacement of the acetabulum into the true pelvis. It usually occurs chronically, as natural forces cause the femoral head to erode through weakened bone.

What is the major impact of the image(s)?

Acute traumatic protrusion jeopardizes nearby structures, including iliac vessels. Traditional pelvic binding increases risk of neurovascular injury, acute hemorrhage, and limb ischemia.

How might this improve emergency medicine practice?

Physicians encountering traumatic acetabular protrusion should avoid pelvic binding, and have low threshold to consult interventional radiology for angiography and possible embolization.

## Figures and Tables

**Image:**
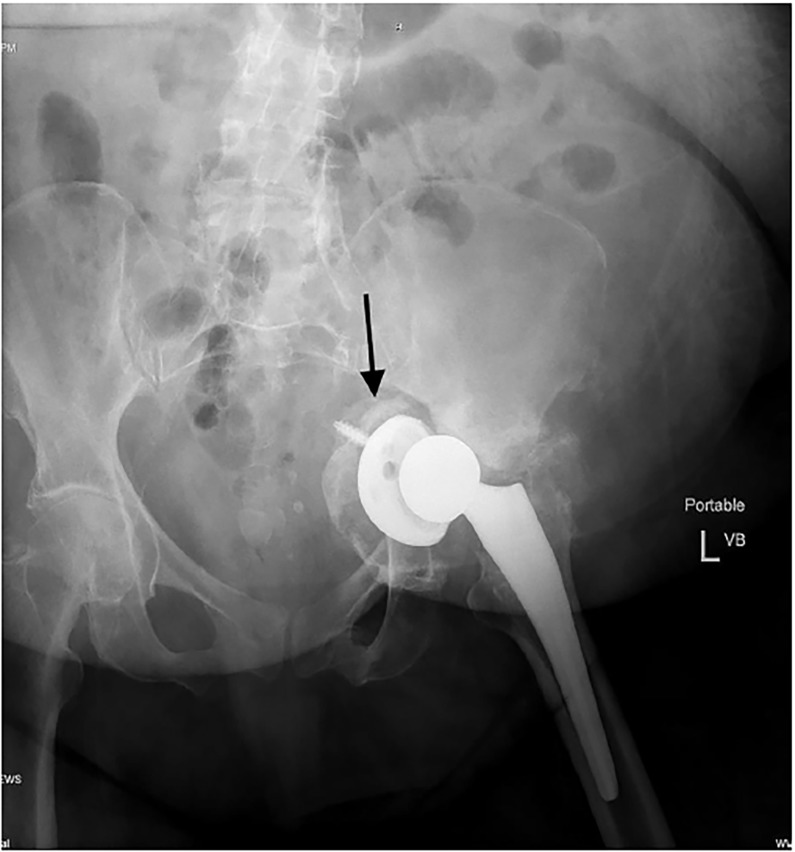
Anterior-posterior radiograph of the pelvis demonstrating medial displacement of the acetabulum and femoral head (arrow) into the lesser pelvis.
